# Notes and News

**Published:** 1897-04-10

**Authors:** 


### NOTES AND NEWS.

(Collected and Communicated.)

Princess Christian has consented to lay the foundation-stone of the new wing for cancer wards at the Middlesex Hospital on July 1st.

The Baroness de Hirsch has contributed £1,000 towards the building of a convalescent home at Brighton in connection with the French Hospital in London.

The Prince of Wales has consented to lay the foundation-stone of the Royal Ophthalmic Hospital now being erected in the City Road. The ceremony is to take place on a date to be fixed after Easter. The cost of the new buildings will be about £85,000.

The Heath Convalescent Home for Working Men at Llanfairfechan, North Wales, has just been opened. It has been established by Messrs. Robert, James, and Arthur Heath, in memory of their father. The Messrs. Heath further intend to partly endow the institution, the management of which will remain in the hands of the donors, to be later placed under a committee. The endowment which the Messrs. Heath propose to provide is to be devoted to providing ten beds for patients from North Staffordshire. The home, which is built in the style of Queen Anne, consists of a three-storied block, and is intended to accommodate 50 patients. The fittings are very complete, and two excellent day-rooms and a bagatelle-room render the comfort of the inmate very complete. All the rooms have open fire-places, and the corridors and some of the rooms are provided with hot-water pipes besides. The situation of the home is admirable. It faces the sea, is only 150 yards from the beach, and is effectually screened by mountains from the east wind.

It is pleasant to note that wherever a colony of British subjects has been formed the same desire to commemorate the Queen's long and prosperous reign is visible. In Rome a meeting has been held at which it has been decided to send a contribution to the Prince of Wales's Fund from the English residents in that city. At Nice a new ward is to be added to the Protestant hospital. Paris is also joining in the commemoration.

The committee of the Hospital for Consumption and Diseases of the Chest at Brompton are asking for funds with which to establish a Country Branch and Convalescent Home. There can be no doubt that such an establishment is a necessary adjunct to an institution dealing with tuberculous cases, and that its inauguration is quite in accordance with modern views as to the treatment of consumption.

The efforts now being made to control the plague in Bombay appear to be having a good effect. The deaths from all causes in that city for the week ending April 2nd numbered 1,099; the reported plague deaths being 481. Great efforts are being made to cleanse and improve the dwellings of the people. During the week ending March 25th 310 dwellings were condemned, 138 were ordered to be altered, 1,028 untiled, 401 dug, 1,210 limewashed, 299 vacated, and 49 demolished. For the week ending April 1st, 83 were condemned, 88 for alterations, 440 untiled, 284 dug, 688 limewashed, and 82 vacated. All this is good and useful work, for it is only by cleansing the city that the plague will be stayed. Various other districts have been attacked, but the arrangements for controlling the disease appear to be now carried out much more systematically than was the case at first.

An appeal has been issued on behalf of the Epileptic Colony established at Chalfont St. Peter's by the National Society for Employment of Epileptics. The claim of the epileptics is founded not merely on the fact that they suffer from a disabling disease, by which they are driven from every form of employment which they may be able to take up, but that in the conditions in which epileptics of the poorer classes commonly live they often sink into insanity, and have to take refuge in asylums. The great preventive of this is the provision of suitable occupation. Many epileptics are strong, and perfectly able to do work when it is provided for them, but their malady prevents them obtaining it, and if they obtain employment soon deprives them of it. The utility of occupation, even as a means of treatment, is well shown by the fact that amongst the numerous candidates waiting for admission to the colony about one in ten drops out of the list every year through insanity or death, whilst of those who have got into the colony, since its establishment nearly three years ago, only one death has occurred, and only one patient has had to be transferred to an asylum. The average number in residence has been twenty-seven, but it is hoped that within a year there will be accommodation for one hundred and sixty epileptics, including men, women, and children. All this will cost money, for which an appeal is now made. There is no doubt about either the importance of the object or the efficiency with which the work is being carried on.

